# Functional outcome, return to work and quality of life in patients with non-aneurysmal subarachnoid hemorrhage

**DOI:** 10.1177/23969873251362012

**Published:** 2025-08-13

**Authors:** Wouter J Dronkers, Menno R Germans, René Post, Bert A Coert, Jonathan M Coutinho, René van den Berg, William Peter Vandertop, Dagmar Verbaan

**Affiliations:** 1 Department of Neurosurgery, Amsterdam UMC Location University of Amsterdam, Amsterdam, The Netherlands; 2 Amsterdam Neuroscience Center, Neurovascular Disease, Amsterdam, The Netherlands; 3 Department of Neurosurgery, University Hospital Zurich, University of Zurich, Zurich, Switzerland; 4 Clinical Neuroscience Center, Zurich, Switzerland; 5 Department of Neurology, Amsterdam UMC Location University of Amsterdam, Amsterdam, The Netherlands; 6 Department of Radiology and Nuclear Medicine, Amsterdam UMC Location University of Amsterdam, Amsterdam, The Netherlands

**Keywords:** Complications, neurology, stroke, subarachnoid hemorrhage, outcome, return to work

## Abstract

**Introduction:**

Non-aneurysmal, non-traumatic subarachnoid hemorrhage (nSAH) refers to cases where a causative aneurysm cannot be identified. We studied 6-months’ outcomes in nSAH patients.

**Patients and methods:**

From a prospective SAH registry of all nSAH patients admitted between 2012 and 2023, relevant complications and outcomes were collected. Functional outcome and return-to-work at 6 months were assessed using the modified Rankin Scale (mRS), quality of life with the EuroQol-5Dimensions (EQ-5D) and Hospital Anxiety and Depression Scale (HADS), and an institutional 14-item questionnaire for assessment of residual symptoms.

**Results:**

325 consecutive nSAH patients were included (192 non-perimesencephalic, non-aneurysmal subarachnoid hemorrhage (NPSAH); 133 perimesencephalic subarachnoid hemorrhage (PMSAH)). 303 (93%; 180 NPSAH and 123 PMSAH) were available at follow-up (7 patients died). Favorable functional outcome (mRS-score 0–2) was reported in 271 (89%) patients and did not differ between NPSAH- and PMSAH. One hundred forty-one (77%) patients returned to work, whereas only 71 (39%) patients reached their previous level of work. PMSAH patients were more likely to return to work (68/96 (71%) NPSAH and 73/87 (84%) PMSAH, respectively, *p* = 0.036). Furthermore, PMSAH patients were more likely to fully return to work (*p* = 0.028). The mean (SD) EQ-5D and EQ-VAS scores were 0.827 (0.184) and 74 (16), respectively. The HADS-A and -D scores were deviant (score > 7 points) in 53 (23%) and 48 (21%) patients, respectively. Only 39 patients (16%) denied experiencing residual symptoms. Increased fatigue (*n* = 164; 68%), increased concentration difficulties (*n* = 130; 54%), and increased forgetfulness (*n* = 121; 50%) were the most frequently reported residual symptoms.

**Discussion and conclusion:**

This study reveals that the majority of nSAH patients reports residual symptoms and did not return to their previous level of work at 6 months follow-up, despite a favorable functional outcome. These findings nuance the perception of a good outcome, as suggested in previous studies, warranting further research on possible rehabilitative interventions and counseling in these patients.

## Graphical abstract

**Graphical abstract:**
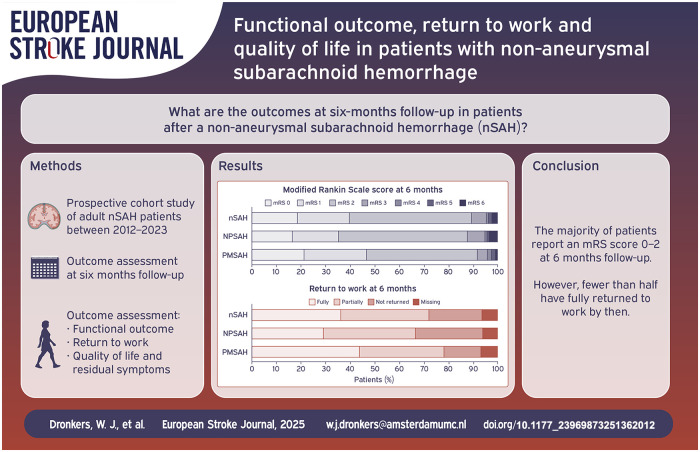

## Introduction

Non-aneurysmal subarachnoid hemorrhage (nSAH) accounts for 15% of all non-traumatic subarachnoid hemorrhages.^1,2^ In contrast to aneurysmal SAH (aSAH), nSAH is characterized by a more favorable clinical course and a better prognosis at 3- and 6-months follow-up.^3^ Among nSAH patients, perimesencephalic subarachnoid hemorrhage (PMSAH) tends to have a better functional outcome than non-perimesencephalic subarachnoid hemorrhage (NPSAH).^4–6^ This difference may be attributed to the substantial variation in hemorrhage volume and, more notably, the occurrence of hydrocephalus, delayed cerebral ischemia, other complications associated with hospitalization, and case fatality.^4,7,8^

The assessment of outcomes in patients following a subarachnoid hemorrhage (SAH) uses established scales such as the modified Rankin Scale (mRS), Glasgow Outcome Scale (GOS) or the GOS extended scale (GOSE).^9^ Favorable outcome is commonly defined as an mRS-score 0–2, a GOS-score 4–5, or a GOSE-score 5–8.^10^ In nSAH patients, the determination of a favorable outcome is often contextualized by a comparison to aSAH.^3,11^ However, these scales frequently place considerable emphasis on physical disability and dependency, while these may not manifest to the same extent in nSAH patients as observed in those with true aneurysmal SAH. Other sequelae, such as the ability to return to work, symptoms of anxiety and depression, and overall decreased quality of life, may also be prevalent and, if overlooked, result in an underestimation, and subsequent under-treatment, of patients, particularly after hospital discharge.^12^

Prospective studies in the nSAH population have mostly focused on functional outcome.^8,13,14^ Studies reporting on residual symptoms, return-to-work after the hemorrhage and quality of life, are relatively sparse, leaving these domains underreported.^15–17^ Moreover, previous studies included relatively small sample sizes, or were conducted in a retrospective fashion with variable, and often short, follow-up times.^18–23^ The aim of the present study is to determine the 6 months outcome in a large prospective cohort of patients after a non-aneurysmal, non-traumatic subarachnoid hemorrhage with a focus on physical disability, return to work, quality of life, and residual symptoms. Additionally, we compare these outcome parameters between NPSAH and PMSAH patients.

## Methods

### Patient population

Data of all consecutive idiopathic SAH patients admitted to the department of Neurosurgery at the Amsterdam University Medical Center, a tertiary referral center for the treatment of SAH patients in the Amsterdam metropolitan area with a total population of approx. 2.5 million people, are systematically recorded in a prospective SAH registry. For this study, we used data from all nSAH patients, admitted from January 2012 up to December 2023. SAH was diagnosed on a non-contrast CT-scan, magnetic resonance imaging (MRI) or by lumbar puncture (LP). Patients were excluded when a CT-angiography (CTA), Digital Subtraction Angiography (DSA), or Magnetic Resonance Angiography (MRA) was not obtained, or could not reliably be assessed. PMSAH was defined as a hemorrhage located in front of the brain stem, mainly in the interpeduncular cistern, with or without extension to the ambient, chiasmal, medial 1/3 part of the horizontal part of the Sylvian cisterns, on a CT-scan performed within 72 h after onset.^24^ Patients with a PMSAH pattern underwent a CTA, however, a DSA was not routinely performed according to local guidelines. All other included nSAH patients were stratified as NPSAH. These patients received both a CTA and a DSA. Patients were excluded when a causative aneurysm for the hemorrhage, or other intracranial vascular pathology including but not limited to arterio-venous malformation, dural arterio-venous fistula, convexity SAH, and cerebral amyloid angiopathy was found. In DSA-negative patients, generally, a repeat DSA or MRI/MRA was performed approx. fourteen days post-hemorrhage. Exceptions for a repeat DSA were made during interdisciplinary neurovascular boards, and included LP-positive SAH patients, and patients with severe atherosclerosis.

Following current (inter)national and local guidelines, nimodipine was administered in all NPSAH patients, but not in PMSAH patients. NPSAH patients were usually admitted at least until the repeat DSA was performed. After diagnosis at our center, PMSAH patients were only admitted to our center if the clinical status of a patient warranted observation in a neurosurgical treatment center. Routine clinical and radiological follow-up in SAH patients was done at 6 months after the hemorrhage.

### Data collection and definitions

Data collection was prospectively performed by a trained research nurse using a predefined Case Report Form (CRF). Castor Electronic Data Capture was used to capture collected data.

Demographic characteristics collected included: sex, age, educational level (no formal education, primary education, secondary vocational education, higher professional education, and academic education), employment and employment type (salaried or self-employed), history of smoking (yes/no), medical history, use of anticoagulant and/or antiplatelet medication (yes/no), premorbid functioning (mRS), World Federation of Neurosurgical Societies (WFNS) score at time of admission to the treatment center, and modified Fisher grade on the first non-contrast CT-scan. Relevant medical history involved intracranial hemorrhage (hemorrhagic cerebrovascular accidents, traumatic hemorrhages), hypertension, cardiovascular disease (ischemic cerebrovascular accident, cardiac events, and other vascular pathologies), diabetes mellitus, and diseases of the central nervous system.

Clinical course characteristics that were collected involved in-hospital mortality, and complications: recurrent bleeding, hydrocephalus (acute and chronic), delayed cerebral ischemia (DCI), seizures, infections (pneumonia, urine tract infection, and meningitis), and delirium. Definitions of the complications can be found in Supplemental Table 1.^25–27^ All results were reported according to the STROBE guidelines.

### Outcome assessment

Outcome assessment is routinely performed by a trained research nurse, either during outpatient clinic appointments or through standardized telephone interviews with follow-up at 6 months after the hemorrhage. If 6 months follow-up was not available, we used the 12 months assessment if present. The research nurse is trained to assess the mRS. The other outcome measures involve questionnaires which are completed by patients themselves.

The mRS (score 0–6) is used to assess functional outcome regarding physical dependency and return to work.^28^ Quality of life is assessed using the EuroQol-5Dimension health questionnaire (EQ-5D; maximum index-score 1000) and EuroQol-visual analogue scale (EQ-VAS; maximum score 100) and the hospital anxiety and depression scale (HADS) (maximum score per category anxiety 21/21 and depression 21/21).^29–31^ For the EQ-5D and EQ-VAS, scores were compared to EQ-5D- and EQ-VAS reference scores for the Dutch population according to age.^29^ Over time, both EQ-5D-3L and EQ-5D-5L were used. Scores were pooled according to the “cross walk method.”^30^ Furthermore, we assess for persistent symptoms such as tiredness, headache, forgetfulness, and anxiety following the hemorrhage by “yes or no” institutional 14-item questionnaire.

### Statistical analysis

Neurological admission status was dichotomized in “good” (WFNS-grade I–III) and “poor” (WFNS-grade IV–V). Functional outcome was dichotomized in “favorable” (mRS score 0–2) and “unfavorable” (mRS score 3–6). Additionally, excellent functional outcome was defined as mRS-score 0–1. Return to work was dichotomized in “yes” and “no.” Patients who were not employed at the time of the hemorrhage (unemployed, stay-at home, retired, or incapacitated) were excluded from the analysis. For HADS and EQ-5D/-VAS scores were reported. For the HADS, a score >7 for each subdomain was suggestive for (potential) anxiety or depression disorder.^31,32^ Finally, the premorbid mRS-score was compared with the mRS-score at 6 months follow-up in which outcomes were defined as “no change,” “higher mRS-score,” and “lower mRS-score” compared to baseline.

Descriptive statistics were reported for all baseline characteristics, the clinical course characteristics, and outcomes at 6-month follow-up for nSAH patients, and stratified per diagnosis (PMSAH and NPSAH). Continuous variables were assessed for normal distribution, with a Shapiro–Wilk value > 0.9 defined as normally distributed. Normally distributed data were reported as means with standard deviations (SD) and as medians with interquartile ranges (IQR) for non-normally distributed data. Student’s *t*-test was done for normally distributed continuous variables and Mann–Whitney *U* test for non-normally distributed continuous variables, respectively. Dichotomous- and categorical variables, and outcome were compared using a Fisher’s exact test. Results with a *p*-value < 0.05 were considered statistically significant. We explored the association between relevant baseline and clinical variables and functional outcome (mRS) and returning to work in nSAH patients using a univariable logistic regression analysis. Variables that were significantly associated with functional outcome or returning to work were included in the multivariable model of that outcome measure. For functional outcome, ordinal regression was used for the univariable analysis. Multinomial logistic regression was used for the multivariable analysis if the independent variables with a significant association with outcome violated the proportional odds assumption (*p* < 0.05). For returning to work, outcome for the univariable and multivariable regression analyses were dichotomous (yes/no), and logistic regression was used. Furthermore, the association between diagnosis (PMSAH and NPSAH) and outcome was assessed using univariable regression analysis. For the association between diagnosis and functional outcome and return to work, analyses corrected for confounders were performed, using ordinal, multinomial logistic, and logistic regression models as described above. To detect confounders, a bivariable analysis was done with demographic variables added individually to the model. All demographic variables were considered potential confounding variables. Demographic variables that changed the crude odds ratio (OR) by more than 10%, were considered confounders. In case confounders were detected and a sufficient number of events in the smallest group of the dependent variable was observed, a multivariable model was built including the main outcomes as dependent variables and confounders and diagnosis group as independent variables. All analyses were done using SPSS statistics, version 28.0.

## Results

### Patient characteristics and clinical course

**Figure 1.**
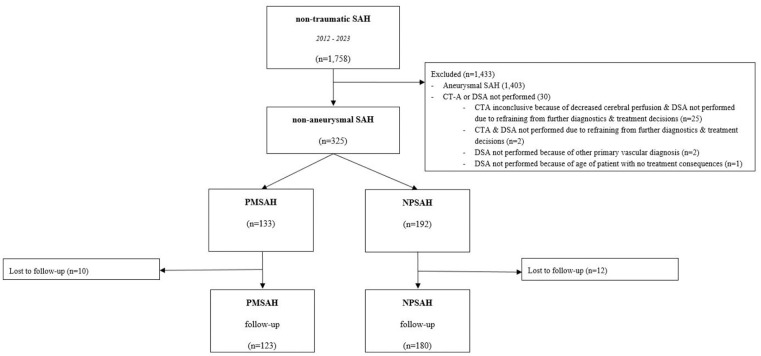
Flowchart. This figure is a flowchart of the included and excluded patients and the number of patients that were followed-up per diagnosis.

A total of 325 nSAH patients (127 (39%) female; mean age 56 years) were included in the present study, comprising 192 (59%) NPSAH and 133 (41%) PMSAH (Figure 1). In DSA negative patients, a repeat DSA and/or MRI/MRA or CTA was performed in 115 (60%) NPSAH patients. Reasons for not performing a repeat DSA were: LP-positive SAH (*n* = 39), the first DSA was performed several days (range 4–14 days) after the initial hemorrhage and was of sufficient quality (*n* = 14), patient specific characteristics (e.g., severe atherosclerosis; *n* = 8), patients’ request (*n* = 1), death prior to repeat imaging (*n* = 5), and unknown reasons (*n* = 9). One-hundred ninety-four (60%) nSAH patients were employed at the time of the hemorrhage (Table 1). Acute hydrocephalus was the most commonly reported complication, affecting 71 patients (22%), with definitive shunting required in 11 patients (15%).

**Table 1.** Baseline characteristics and clinical course of 325 nSAH patients.

Variable	nSAH, *n* = 325	NPSAH, *n* = 192	PMSAH, *n* = 133	*p*-Value
Age, years, mean (SD)*	56 (11)	57 (11)	54 (11)	0.040
Sex, female	127 (39)	71 (37)	56 (42)	0.358
Medical history				
Hypertension	77 (24)	57 (30)	20 (15)	0.002
Cardiovascular disease	53 (16)	41 (21)	12 (9)	0.003
Central nervous system	20 (6)	14 (7)	6 (4)	0.355
Intracranial hemorrhage	3 (1)	3 (2)	0 (-)	0.272
Diabetes mellitus	25 (8)	20 (10)	5 (4)	0.033
Anxiety	3 (1)	0 (-)	3 (2)	0.070
Depression	14 (4)	7 (4)	7 (5)	0.583
Psychiatric, other	6 (2)	5 (3)	1 (1)	0.41
Anticoagulation/-platelet therapy	49 (15)	37 (19)	12 (9)	0.012
Smoking	164 (50)	103 (54)	61 (46)	0.109
Premorbid functioning				
mRS-score > 0^†^	69 (23)	41 (24)	28 (22)	0.782
Education (higher education)	133 (40)	72 (38)	61 (46)	0.231
Employment status				
Employed	194 (60)	102 (53)	92 (69)	0.006
Not employed				
Unemployed	16 (5)	10 (5)	6 (5)	0.800
Retired	70 (22)	48 (25)	22 (16)	0.054
House-wife and -man	11 (3)	6 (3)	5 (4)	0.767
Incapacitated	15 (5)	13 (7)	2 (1)	0.029
Missing (employment status)	19 (6)	13 (7)	6 (5)	NA
Employment type (salaried)	146/194	83/102	63/92	0.043
Admission characteristics				
WFNS-grade I–III	313 (96)	181 (94)	132 (99)	0.032
mFisher 0	46 (14)	46 (24)	NA	NA
mFisher 3 or 4	248 (76)	132 (69)	116 (87)	<0.001
Complication				
Acute hydrocephalus	71 (22)	62 (32)	9 (7)	<0.001
Shunt dependent^‡^	11 (15)	10 (16)	1 (11)	1.000
Rebleeding	3 (1)	3 (2)	0 (-)	0.272
DCI	5 (2)	5 (3)	0 (-)	0.081
Meningitis	17 (5)	16 (8)	1 (1)	0.002
Seizures	10 (3)	10 (6)	0 (-)	0.006
Delirium	8 (2)	7 (4)	1 (1)	0.148
Pneumonia	6 (2)	6 (3)	0 (-)	0.085
Urine tract disease	5 (2)	4 (2)	1 (1)	0.652
Sepsis	1 (>1)	1 (1)	0 (-)	1.000

nSAH: non-aneurysmal subarachnoid hemorrhage; NPSAH: non-perimesencephalic non-aneurysmal subarachnoid hemorrhage; PMSAH: perimesencephalic non-aneurysmal subarachnoid hemorrhage; *n*: number; SD: standard deviation; Na: not applicable; mFisher: modified Fisher; DCI: delayed cerebral ischemia; WFNS: world federation of neurological surgeons; N/A: not applicable.

Data are presented as numbers (%), unless otherwise reported. The Fisher exact test is used unless otherwise specified.

* Student’ *t*-test.

† Calculated over 297(171 NPSAH and 126 PMSAH patients).

‡ Fraction of hydrocephalus patients.

### Outcome nSAH

Follow-up data were available for 303 patients (93%), comprising 180 (94%) and 123 (92%) patients of the NPSAH and PMSAH population, respectively (Table 2). Reasons for missing follow-up at 6 months were relocation to another region or country (*n* = 5), at patients’ request (*n* = 5), and unknown (*n* = 12). Seven (2%) patients died between hemorrhage and follow-up, all during hospital stay. The reported causes of death included refraining from further treatment in cases of persisting coma due to the hemorrhage (*n* = 4), refraining from further treatment in an elderly patient with hydrocephalus (*n* = 1), due to respiratory failure (*n* = 1), and multi-organ failure (*n* = 1). In 11 patients (4%), a 12 months follow-up was used due to absence of a 6 months assessment.

**Table 2.** Outcome at 6 months’ follow-up in 303 nSAH patients.

Variable	nSAH	NPSAH	PMSAH	*p*-Value
Follow-up	303 (93)	180 (94)	123 (92)	0.660
Loss to follow/up	22 (7)	12 (6)	10 (7)	NA
Follow-up time, median (Q1/Q3), months	6 (5.9/6.4)	5.9 (5.8/6.3)	6.2 (6.0/6.5)	0.787
Functional outcome, mRS				
All-cause mortality at 6 months (mRS 6)	7 (2)	6 (3)	1 (1)	0.246
Favorable (mRS-score 0–2)	271 (89)	157 (87)	114 (93)	0.182
Excellent (mRS-score 0–1)	120 (40)	64 (36)	56 (46)	0.094
Functional outcome, compared to baseline*				
No change	79 (27)	45 (26)	34 (28)	0.804
Higher mRS-score	204 (69)	122 (70)	82 (68)	
Lower mRS-score	12 (4)	8 (5)	4 (3)	
Functional outcome, return to work^†^				
Returned to work	141 (77)	68 (71)	73 (84)	0.036
a. Fully returned to work	71 (39)	30 (31)	41 (47)	0.028
b. Partially returned to work	70 (38)	38 (40)	32 (37)	0.697
Not returned to work	42 (23)	28 (29)	14 (16)	0.036
Quality of life				
EQ-5D, mean score (SD)^‡^	0.827 (0.184)	0.825 (0.183)	0.830 (0.187)	0.848
EQ-VAS, mean score (SD)^§^	74 (16)	74 (17)	75 (15)	0.363
HADS total, mean score (SD)^||^	9.4 (8)	9.5 (8)	8.9 (8)	0.424
Anxiety, mean score (SD)	5.2 (4)	4.9 (4)	5.5 (4)	0.238
Anxiety deviant (score > 7)	53 (23)	36 (26)	17 (18)	0.172
Depression, mean score (SD)	4.2 (4.2)	4.3 (4)	4.0 (4)	0.658
Depression deviant (score > 7)	49 (21)	32 (23)	17 (18)	0.394
Residual symptoms^¶^				
One or more residual symptoms	202 (84)	125 (86)	77 (81)	0.347

SD: standard deviation; IQR: interquartile range; mRS: modified Rankin Scale; HADS: hospital anxiety and depression scale; EQ-5D: EuroQol-5 dimensions.

Data are presented as numbers (%), unless otherwise reported. The Fisher exact test is used unless otherwise specified.

* Calculated over 295 (174 NPSAH and 121 PMSAH) patients.

† Calculated over 183 (96 NPSAH and 87 PMSAH) patients of which the employment status was known after the hemorrhage.

‡ Calculated over 240 (141 NPSAH and 99 PMSAH) patients.

§ Calculated over 231 (139 NPSAH and 92 PMSAH) patients.

|| Calculated over 235 (141 NPSAH and 94 PMSAH) patients.

¶ Calculated over 241 (146 NPSAH and 95 PMSAH) patients.

**Figure 2.**
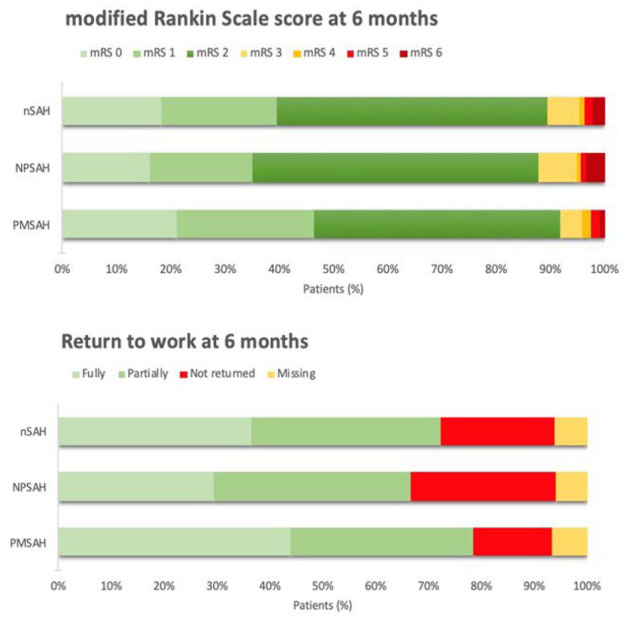
Functional outcome at 6 months follow-up. This figure provides an overview of the mRS-scores and return to work rates at 6 months follow-up for nSAH, NPSAH, and PMSAH.

A favorable functional outcome (mRS-score 0–2) was reported in 271/303 patients (89%) at a median (IQR) follow-up duration of six (5.9–6.4) months (Figure 2). Excellent outcome (mRS-score 0–1) was reported in 120 patients (40%). In 204–295 (67%) patients, the mRS-score worsened compared to their baseline score. At 6 months follow-up, 141/183 (77%) patients returned to work (return to work was missing in 12 patients who were lost to follow-up; Figure 2). Fully returning to the previous level (same position and workload) of work was reported in 71/183 patients (39%).

Symptoms of increased anxiety (HADS-A) and depression (HADS-D) were present in 53/234 (23%) and 49/235 (21%) patients. In 38/234 patients (16%), both HADS-A and -D were increased. For the HADS-A, all except for one patient were not known with a history of anxiety. For the HADS-D, all except for five patients had no history of depression. The other patients with a history of depression did not report scores above the threshold at follow-up. Residual symptoms, based on the institutional PROMS 14-item questionnaire, were present in 202/241 patients (84%). Only 39 patients (16%) denied experiencing residual symptoms. The most frequently reported symptoms included earlier fatigue (*n* = 164; 68%), increased difficulties in concentration (*n* = 130; 54%), and increased forgetfulness (*n* = 121; 50%; Supplement Table 2).

### NSAH uni- and multi-variable regression analyses

Univariable ordinal regression analysis for functional outcome in nSAH revealed sex (male; OR: 1.82; *p* = 0.007), premorbid mRS-score 0 (OR: 2.82; *p* < 0.001), higher education (OR: 1.96; *p* = 0.004), WFNS-grade I–III (OR: 9.5; *p* < 0.001), and absence of hydrocephalus (OR: 3.37; *p* < 0.001) were associated with a more favorable functional outcome (lower mRS-score) at follow-up (Table 3). The multivariable model revealed that a premorbid mRS-score 0 (aOR: 2.24; *p* = 0.006), higher education (aOR: 1.64; *p* = 0.037), and absence of hydrocephalus (aOR: 3.21; *p* < 0.001) were significantly associated with better functional outcome.

**Table 3.** Uni and multivariable regression analysis.

Factor (group)	Univariable regression analysis			Multivariable regression analysis		
	OR	95% CI	*p*-Value	aOR	95% CI	*p*-Value
mRS-score						
Diagnosis (PMSAH)	1.51	1.02–2.32	0.061	Na		
Sex (male)	1.82	1.18–2.86	0.007	1.50	0.92–1.08	0.100
Age (18–64)	1.52	0.93–2.50	0.092	Na		
Premorbid functioning (mRS-score 0)	2.82	1.62–4.89	<0.001	2.24	1.26–3.96	0.006
Education (higher education)	1.96	1.23–3.03	0.004	1.64	1.03–2.63	0.037
Employment type (self-employed)	1.59	0.82–3.03	0.170	Na		
WFNS-grade (I–III)	9.5	2.9–31.3	<0.001	1.52	0.28–8.71	0.625
mFisher 0–2	1.36	0.83–2.22	0.229	Na		
Hydrocephalus (no)	3.37	1.94–5.85	<0.001	3.21	1.71–6.06	<0.001
Returning to work						
Diagnosis (PMSAH)	2.15	1.04–4.42	0.038	1.32	0.57–3.05	0.514
Sex (male)	2.38	1.17–4.83	0.017	2.22	1.01–4.91	0.048
Age (18–64)	2.09	0.48–9.15	0.327	Na		
Premorbid functioning (mRS-score 0)	1.41	0.57–3.47	0.461	Na		
Education (higher education)	3.48	1.62–7.46	0.001	3.42	1.53–7.6	0.003
Employment type (self-employed)	2.86	1.05–7.80	0.040	2.03	0.70–5.95	0.195
WFNS-grade (I–III)	Na			Na		
mFisher 0–2	1.00	0.46–2.20	0.995	Na		
Hydrocephalus (no)	4.11	1.89–8.95	<0.001	3.36	1.36–8.29	0.009

OR: odds ratio; aOR: adjusted odds ratio; CI: confidence interval; Na: not applicable.

Interpretation: an OR > 1 represents an increased likelihood for better functional outcome (lower mRS-score). For returning to work an OR > 1 represents an increased likelihood for returning to work. No patients with WFNS-grade IV–V returned to work. Therefore, an OR could not be calculated.

Univariable logistic regression analysis for returning to work revealed that diagnosis (PMSAH; OR: 2.15; *p* = 0.038), sex (male; OR: 2.38; *p* = 0.017), higher education (OR: 3.48; *p* = 0.001), self-employed job status (OR: 2.86; *p* = 0.040), and absence of hydrocephalus (OR: 4.11; *p* < 0.001) were associated with an increased likelihood to return to work (Table 3). The multivariable model revealed that sex (male; aOR: 2.22; *p* = 0.048), higher education (aOR 3.42; *p* = 0.003) and absence of hydrocephalus (aOR: 3.52; *p* = 0.006) were independently associated with an increased likelihood to return to work.

### NPSAH versus PMSAH

NPSAH patients were older, suffered from various comorbidities and were less often employed at the time of the hemorrhage (Table 1). Acute hydrocephalus was reported in 62 NPSAH patients (32%) and 9 PMSAH patients (7%; *p* < 0.001). NPSAH patients were more likely to develop a meningitis (*p* = 0.002). Rebleeding and delayed cerebral ischemia exclusively occurred in NPSAH patients (Table 2).

In the univariable model, diagnosis was not significantly associated with functional outcome (PMSAH; OR: 1.51; 95% CI: 1.02–2.32; 0.061). Confounders for the association between diagnosis and functional outcome were cardiovascular disease, the use of anticoagulant or platelet therapy, educational level, employment type, and premorbid functioning. After correction, the multivariable model revealed that diagnosis and outcome were significantly associated with PMSAH patients being more likely to report better functional outcome (aOR: 2.02; 95% CI: 1.09–3.76; 0.025).

In the univariable model, PMSAH patients were more likely to return to work (OR: 2.15; 95% CI: 1.04–4.42). Furthermore, PMSAH patients were more likely to fully return to work (30 (31%) NPSAH, 41 (47%) PMSAH, respectively, *p* = 0.028). Educational level and employment type were both confounders and after correction in a multivariable model, diagnosis and returning to work were not significantly associated anymore (aOR: 1.73; 95% CI: 0.79–3.71; *p* = 0.172). No differences were found in EQ-5D, EQ-VAS, and HADS scores. No significant differences were reported regarding residual symptoms (Supplement Table 2). Subgroup analysis leaving out the 46 NPSAH mFisher 0 (LP-positive SAH) showed no differences in functional outcome or quality of life.

## Discussion

This large prospective, single-center, observational study of non-aneurysmal, non-traumatic subarachnoid hemorrhage patients reveals that the majority of patients did not return to their previous level of work despite that most patients reported a good outcome (mRS 0–2) at 6 months follow-up. Furthermore, this study shows that residual symptoms, such as increased fatigue and concentration difficulties, are commonly reported. These findings may nuance the perception of what has been previously been supposed as good outcome in these patients.

Outcome in patients after SAH has been extensively studied, often focusing on physical dependency.^5,11,13^ The assessment of functional outcome in the present study was done using the mRS. Alike previous studies, the majority of patients achieved a favorable (mRS-score 0–2) outcome.^6^ The majority of nSAH patients in this study had an mRS-score 2, reflecting slight disability but not returning to all previous daily activities. The standardized mRS telephone interview assesses whether patients were employed at the time of the hemorrhage, and whether they experience problems with their work at follow-up. Patients who do not return to their previous level of work or do not return to work at all are reported as an mRS-score 2. In our multivariable model, we found that patients who were male, had higher education, or those without hydrocephalus were more likely to return to work. Overall, the majority of the patients who were employed at the time of the hemorrhage did not fully return to work, or did not (yet) return to work at all at 6 months follow-up. This prompts the question of whether resuming prior daily activities, including returning to work, can be used as a criterion for favorable outcome, and how an “excellent outcome” might be defined. In previous studies, the outcome for nSAH patients may have been considered excellent by comparing it to aSAH in terms of physical dependency and mortality.^6,9^ However, studies comparing aSAH and nSAH cohorts remain sparse.^3,33–35^ For aSAH, an mRS-score 0–2 or even 0–3 is a commonly used, an broadly accepted cut-off for defining favorable outcome.^9,36^ For nSAH, a favorable (functional) outcome may better be defined as mRS-score 0–1, as argued by Patel et al.^37^ In the present study, about 40% of nSAH patients reported an mRS-score 0–1. From this perspective, fewer than half of the patients report an “excellent outcome” at 6 months follow-up. Furthermore, the majority of the patients report one or more residual symptoms, which implicates that outcome involves more than (physical) dependency. Presence of these symptoms at follow-up may to some extent explain why patients did not (fully) return to their work or resuming former daily activities, and the proportion of patients reporting a decreased quality of life, or symptoms of anxiety and/or depression. These findings nuance the relatively benign clinical course of nSAH patients.

As a personalized post-hemorrhage support system is becoming increasingly important, the findings of the present study could contribute for the counselling of these patients. In this regard, early recognition of deficits during follow-up in patients may increase the attention for implementing additional interventions. For the follow-up of SAH patients at our center, we use the 14-item questionnaire to address various symptoms that have been previously mentioned by patients underlining the importance of using patient-reported outcome measures (PROMS). The use of PROMS has been previously studied in stroke.^38–42^ It was found that although patients may report a favorable functional outcome on the mRS-score, patients could still report other complaints resulting in an overall unfavorable outcome.^38,42^ By using PROMS as a screening tool during follow-up, nSAH patients that may require additional follow-up or care after discharge may be more easily recognized and specific interventions could be initiated accordingly. One such intervention could be rehabilitation. Previous studies have already addressed the possible positive influence of rehabilitation on recovery in nSAH patients.^43,44^ Findings of the present study regarding not (fully) returning to work and presence of residual symptoms underline the possible relevance of rehabilitation in these patients and warrants further investigation in larger prospective studies.

The present study holds some strengths and limitations. Strengths involve the prospective nature of the study including a structured follow-up assessment by a trained research nurse as well as the large sample size compared to previous (prospective) studies.^8,13–15,18–23^ Furthermore, less than 10% of all patients were lost to follow-up. In the present study, we used validated (mRS, EQ-5D, EQ-VAS, and HADS) and non-validated PROMS (14-item questionnaire) tools for assessing outcome in multiple dimensions. Although the research nurse was not blinded for diagnosis, we think that the extended, standardized form of the mRS, which was used to assess functional outcome, decreased the likelihood for observer or confirmation bias. Finally, the overall generalizability of the data presented in the present study is expected to be high for developed countries since the standard of care is comparable within these regions. Several limitations should be noted. First, the number of missing data for the HADS, EQ-5D, and the 14-item residual symptoms questionnaire. A possible explanation is that follow-up, especially since the COVID-19 pandemic, was increasingly done through telephone instead of in-person consults. This could have made assessment of the questionnaires more challenging and therefore lead to a higher likelihood of missing data and introduced bias in the reported findings. Selection bias may be present if patients are physically not able to fill out questionnaires, resulting in an overestimation of good outcome in the study population. Second, the absence of a neuropsychological evaluation to further explore the reported residual symptoms, as well as the assessment of these symptoms by a questionnaire, are other limitations which could be a direction for further research. Third, PMSAH patients were not routinely admitted to our center after diagnosis and adverse events at the referring hospital may not have been communicated to our center. However, since our center serves as the main tertiary referral center for SAH care, chances of adverse events not being communicated to our center are expected to be small. Finally, the lack of information regarding the reason of not (fully) returning to work can be considered another limitation. It remains unclear whether not fully returning to work was due the physical and/or psychological impairments after the hemorrhage or whether it was the patients’ own decision to ease off after the hemorrhage and to retire (earlier).

## Conclusion

The majority of patients did not yet (fully) return to their previous level of work, despite reporting a favorable mRS 0–2 score, at 6 months follow-up after non-aneurysmal, non-traumatic subarachnoid hemorrhage. Moreover, most patients report long-lasting residual symptoms. These findings nuance the proposed “good outcome” in non-aneurysmal subarachnoid hemorrhage patients and warrants further research. This could focus on prospectively assessing outcome using PROMS in larger sample sizes at long-term follow-up as well as studying the influence of rehabilitation on recovery and returning to work.

## Supplemental Material

sj-docx-1-eso-10.1177_23969873251362012 – Supplemental material for Functional outcome, return to work and quality of life in patients with non-aneurysmal subarachnoid hemorrhage Supplemental material, sj-docx-1-eso-10.1177_23969873251362012 for Functional outcome, return to work and quality of life in patients with non-aneurysmal subarachnoid hemorrhage by Wouter J Dronkers, Menno R Germans, René Post, Bert A Coert, Jonathan M Coutinho, René van den Berg, William Peter Vandertop and Dagmar Verbaan in European Stroke Journal
