# Supplementary material for: Functional outcome, return to work and quality of life in patients with non-aneurysmal subarachnoid hemorrhage
Source: Eur Stroke J. 2025 Aug 13:23969873251362012. Online ahead of print. doi: 10.1177/23969873251362012 (PMC12350299; doi:10.1177/23969873251362012)
Supplement: sj-docx-1-eso-10.1177_23969873251362012 – Supplemental material for Functional outcome, return to work and quality of life in patients with non-aneurysmal subarachnoid hemorrhage [file sj-docx-1-eso-10.1177_23969873251362012.docx]

**Supplement tables**

**Supplement table 1. Definitions of various clinical terms**

| **Clinical term** | **Definition** |
| --- | --- |
| **Recurrent bleeding** (rebleeding) | A second (or third etc.) bleeding after the initial bleeding. Rebleeding was either diagnosed with a non-contrast CT-scan or in case of high clinical suspicion (e.g., acute clinical deterioration combined with abrupt increase in blood pressure, bradycardia, respiratory alterations, or the appearance of a sudden increase in production of CSF with fresh blood through ventricular drainage. |
| **Acute hydrocephalus**  **Chronic hydrocephalus** | Enlarged ventricles on imaging, assessed by an experienced neuro-radiologist, or by increased intracranial pressure diagnosed by lumbar puncture or ventricular catheter placement or by increased intracranial pressure diagnosed through a lumbar puncture or ventricular catheter placement.  Requiring definitive CSF-shunting. |
| **Delayed cerebral ischemia** (DCI) | The occurrence of focal neurological impairment (such as hemiparesis, aphasia, apraxia, hemianopia, or neglect), or a decreased of at least 2 points on the Glasgow Coma Scale. This should last for at least 1 hour, is not apparent immediately after aneurysm occlusion, and cannot be attributed to other causes by means of clinical assessment, CT or magnetic imaging scanning of the brain, and appropriate laboratory studies. |
| **Seizures** | Clinical appearance of rhythmic tonic or clonic movements for which anti-epileptic drugs were started or by electroencephalography (EEG). |
| **Pneumonia** | Clinical symptoms followed by a positive culture the microbiological analysis of sputum or a consolidation on the chest X-ray or when antibiotics were prescribed specifically for this indication. |
| **Urinary tract infection** | Clinical symptoms followed by a positive culture based on the microbiological analysis of urine or leukocytosis or bacteremia in the urine sediment. |
| **Meningitis** | Clinical symptoms followed by a positive culture based on the microbiological analysis of CSF or when antibiotics were prescribed specifically for this indication. |
| **Delirium** | In case of treatment with haloperidol and patients had a delirium observation screening (DOS) score >4 or if the Richmond Agitation Sedation Scale (RASS) was scored between +4 and -4. |

**Supplement table 2. 14-item questionnaire**

| **Questionnaire item** | **nSAH** | **NPSAH** | **PMSAH** | *p-value* |
| --- | --- | --- | --- | --- |
| Q1. Increased fatigue *** | 164 (68) | 104 (71) | 60 (63) | 0.189 |
| Q2. More sensitive for loud noises *** | 111 (46) | 61 (42) | 50 (53) | 0.099 |
| Q3. Increased forgetfulness *** | 121 (50) | 77 (53) | 44 (46) | 0.330 |
| Q4. Increased concentration difficulties *** | 130 (54) | 77 (53) | 53 (55) | 0.643 |
| Q5. Increased headache *** | 100 (42) | 61 (42) | 39 (41) | 0.911 |
| Q6. Increased insomnia † | 50 (21) | 31 (21) | 19 (20) | 0.829 |
| Q7. Increased daytime sleepiness *** | 112 (47) | 72 (49) | 40 (42) | 0.273 |
| Q8. Worse or blurred vision ‡ | 66 (28) | 38 (26) | 28 (29) | 0.602 |
| Q9. Increased dizziness *** | 67 (28) | 42 (29) | 25 (26) | 0.678 |
| Q10. Increased excitable § | 92 (38) | 54 (38) | 38 (40) | 0.593 |
| Q11. Increased anxious feelings *** | 84 (35) | 55 (38) | 29 (31) | 0.255 |
| Q12. Increased gloominess *** | 60 (25) | 39 (27) | 21 (22) | 0.419 |
| Q13. Increased balance problems *** | 57 (24) | 36 (25) | 21 (22) | 0.649 |
| Q14. Increased lack of initiative \|\| | 67 (28) | 41 (29) | 26 (28) | 0.827 |

** Calculated over 241 (146 NPSAH and 95 PMSAH) patients.* † *Calculated over 239 (145 NPSAH and 94 PMSAH) patients.* ‡ *Calculated over 239 (144 NPSAH and 95 PMSAH) patients.* § *Calculated over 240 (146 NPSAH and 94 PMSAH) patients.* || *Calculated over 238 (143 NPSAH and 95 PMSAH) patients.*
